# Efficacy of mandibular advancement device in the treatment of obstructive sleep apnea syndrome: A randomized controlled crossover clinical trial

**DOI:** 10.4317/medoral.20649

**Published:** 2015-08-04

**Authors:** Joaquín Durán-Cantolla, Rafael Crovetto-Martínez, Mohammad-Hamdan Alkhraisat, Miguel Crovetto, Antonio Municio, Ramón Kutz, Felipe Aizpuru, Erika Miranda, Eduardo Anitua

**Affiliations:** 1 MD, PhD. Sleep Disorders Unit. Hospital Universitario Araba, Vitoria-Gasteiz. Spain; 2 MD, PhD. MD, PhD. BS Research Service. BioAraba Institute. Hospital Universitario Araba, Vitoria-Gasteiz. Spain; 3 MD, PhD. Faculty of Medicine. University of Basque Country (UPV/EHU). Spain; 4 MD, PhD. Ciber of Respiratory Diseases (CibeRes). ISCIII, Madrid. Spain; 5 MD, PhD. DDS PhD. MD, DDS, PhD. Eduardo Anitua Clinic and Foundation. Vitoria-Gasteiz. Spain; 6 MD, PhD. Department of Stomatology II, Faculty of Medicine and Dentistry, UFI 11/25, University of the Basque Country/EHU Spain; 7 MD, PhD. Servicio ORL. Hospital de Basurto. Bilbao. Vizcaya. Spain; 8 MD. Antonio Municio. Servicio de ORL. Hospital de Curces. Baracaldo. Vizcaya; 9 MD. Ramón Kutz. Clínica Estomatológica. Vitoria

## Abstract

**Background:**

Evaluation of the efficacy and safety of a mandibular advancement device (MAD) (KlearwayTM) in the treatment of mild-to-moderate obstructive sleep apnea and chronic roncopathy.

**Material and Methods:**

A randomized, placebo-controlled, double blinded, and crossover clinical trial was conducted. Placebo device (PD) defined as a splint in the centric occlusion that did not induce a mandibular advancement served as a control. The mandible was advanced to the maximum tolerable distance or to a minimum of 65% of the maximum protrusion. After each sequence of treatment, patients were assessed by questionnaires, conventional polysomnography, and objective measurement of snoring at the patient’s own home.

**Results:**

Forty two patients participated in the study and 38 completed the study. Patients mean age was 46 ±9 years and the 79% were males. The mean mandibular advancement was 8.6 ±2.8 mm. Patients used the MAD and the PD for 6.4 +2.4 hours and 6.2 +2.0 hours, respectively. Secondary effects (mostly mild) occurred in the 85.7% and the 86.8% of the users of MAD and PD, respectively. The MAD induced a decrease in the apnea-hypopnea index (AHI) from 15.3 +10.2 to 11.9 +15.5. The 50% reduction in the AHI was achieved in the 46.2% and the 18.4% of the patients treated with MAD and PD, respectively. The use of the MAD induced a reduction in the AHI by 3.4 +15.9 while the PD induced an increase by 10.6 +26.1. The subjective evaluation of the roncopathy indicated an improvement by the MAD and an increase in the perceptive quality of sleep. However, the objective evaluation of the roncopathy did not show significant improvements.

**Conclusions:**

The use of MAD is efficient to reduce the AHI and improve subjectively the roncopathy. MAD could be considered in the treatment of mild-to-moderate OSA and chronic roncopathy.

**Key words:**Obstructive sleep apnea (OSA), mandibular advance device, treatment, efficacy, clinical assay.

## Introduction

The obstructive sleep apnea (OSA) is a prevalent disease that affects in its severe form 2-8% of the general population. It is estimated that more than 20% of the population has an apnea-hypopnea index (AHI) value that is ≥ 5 (1,2). Clinical symptoms of OSA include somnolence, neuropsychiatric and cardio-respiratory disorders. These complications result from anatomical and functional alterations of the upper airway where repetitive episodes of obstruction during sleep provoke oxihemoglobin desaturation and temporal arouses that lead to a none-reparative sleep (3).

Many studies have established OSA as a risk factor for arterial hypertension and traffic accidents (2,4-6). The presence of OSA has also been related to cardiovascular and cerebrovascular complications (5,7,8). Even more, higher mortality has been reported among patients with OSA (9,10). In spite of all these consequences, OSA is still not adequately managed. Only 10% of the population with OSA are diagnosed and treated (3,11,12).

The administration of a continuous positive airway pressure (CPAP) is an efficient, cost-effective treatment of OSA (3,13,14). There is a total agreement to indicate this treatment in patients with AHI > 30 who suffer from complications related to the OSA (3). However, the indication of the CPAP in patients with mild-to-moderate OSA (AHI = 5-29) is not that clear (3,14,15).

The mandibular advancement device (MAD) is considered as an alternative to the CPAP (3). This device, also designed to alleviate and treat roncopathy, provokes the protrusion of the mandible in order to elevate and advance both the hyoid bone and the tongue. By this, the volume of the upper airway is increased making less likely the passage narrowing or collapse (16). Several studies have recommended the treatment of mild-to-moderate OSA by MAD and have also indicated its use in patients with severe OSA intolerable to the CPAP (3,17,18). However, there is still a need for randomized controlled clinical trials to establish with more precision the indications and the efficacy of the MAD in the treatment of OSA.

In this study, a randomized controlled crossover clinical trial has been performed to evaluate the efficacy and safety of mandibular advancement device in the treatment of patients with mild-to-moderate OSA. A placebo device (PD) has been used as a control.

## Material and Methods

* Patients 

Randomized, placebo-controlled, double blinded, and crossover clinical trial was conducted at the Interdisciplinary Unit of Sleep Disorders of Alava University Hospital. The study protocol and informed consent, in full accordance with the ethical principles of the Declaration of Helsinki of 1975, as revisited in 2000, were approved by the ethical committee of University Hospital of Álava (Vitoria-Gasteiz, Spain).

Patients were consecutively selected from adult subjects referred due to a clinical suspicion of OSA. Patients from both sexes were eligible to participate in this study and were selected according to the following inclusion criteria:

- Age higher than 18 years, 

- Presence of chronic snoring. A patient is considered as chronic snorer if his/her bed mate/roommate reported to snore more than 5 days per week and this is corroborated by a respiratory polygraphy performed in the patient’s own home. The result of the respiratory polygraphy should indicate the presence of snoring during at least 30% of the nocturnal period.

- Confirmed diagnosis of mild-to-moderate OSA (5 ≤ AHI < 30) by polysomnography (PSG).

- Have a roommate or bed mate to submit information.

Patients were excluded according to the following exclusion criteria:

- High-risk professions and/or controlling dangerous machines.

- Moderate or severe somnolence during day time.

- Coronary cardiopathy, acute vascular disease (less than three months), chronic and severe obstructive pulmonary disease, and chronic treatment with theophyllines.

- Temporo-mandibular joint problems or periodontitis.

- Mandibular protrusion capacity less than 6 mm and/or less than 10 teeth in each jaw.

- Severe cognitive disorders and/or patients whose answers to the questionnaires will be altered by chronic and severe diseases.

- Pregnancy (since the third month of pregnancy to 3 months after birth delivery).

**Figure 1:**
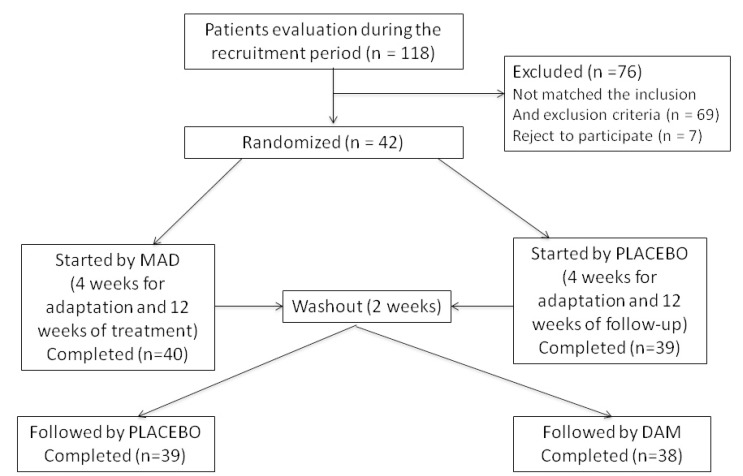
Study flow chart.

A total of 118 patients were screened, of whom 76 were not eligible: 62 did not meet the inclusion criteria or had one or more of the exclusion criteria, and 7 declined to participate (Fig. 1).

Forty-two patients were randomly assigned to receive two possible sequences of treatment (MAD or placebo device (PD) according to a computer-generated randomization schedule. To achieve a double-blinded study, professionals not related to the interventions and opaque-sealed envelopes were adopted. The dentists and ENT surgeons, responsible of device fabrication, adaptation, and following up of the patients, had no contact during the clinical trial with the professionals who performed the sleep study. All the controls and the results of the tests were code identified and were only known by a one professional who did not participate in the clinical trial.

Initially, each patient were subjected to a period of 2 weeks without any treatment, followed by 4 weeks of adaptation and standardization of the device (MAD or PD), and 12 weeks of treatment (Fig. 1). Once this period was finished, patients were switched to use the other device following the same protocol as described above (Fig. 1).

Mandibular advancement device (MAD): The commercial device KlearwayTM (University of British Columbia, Vancouver, Canada) was used. The fabrication of the device was made on model casts of both jaws and was adapted to the patient’s mouth by a dentist with the objective to achieve a sufficient and tolerable mandibular advancement, being at least 65% of the maximum protrusion capacity of the mandible. This phase may need more than one visit to the dentist and had a period of 4 weeks at maximum.

Placebo device (PD): The placebo device was the same KlearwayTM device but in centric occlusion and did not provoke mandibular advancement. The dentist assured the absence of mandibular advancement and alteration to the TMJ position. The reference point was jaw position at the TMJ level in rest as measured by cephalometry. The PD adaptation may need more than one visit to the dentist and had a period of 4 weeks at maximum.

* Primary outcome

The primary outcome was the apnea hypopnea index (AHI) that was measured by a conventional polysomnography (PSG). The PSG study was realized at the Interdisciplinary Unit of Sleep Disorders of Alava University Hospital. The PSG study was performed before the study and after the 12 weeks of the treatment with each device (MAD and PD).

All sleep studies were performed with PSG (Alice 3 Health dyne system) according to the standard parameters of electroencephalogram (C3-A2, C4-A1), electrooculogram, submentonian and tibial electromyogram, electrocardiogram (modified V2), respiratory effort (thorax and abdominal resistance), air flow (nasal and oral thermistor), Oxygen saturation (cutaneous pulsioximetry with a finger probe (Palco laboratories P-340) and snore microphone. The PSG study was manually interpreted in periods of 30 seconds according to the criteria of the American Academy of Sleep Studies (19) and following the guidelines of the Spanish Society of Pneumology and Thoracic Surgery (SEPAR) (3). The minimum time of recording was 6 hours and the minimum time of sleep was 180 minutes. The following definition of the respiratory variables were used.

- Apnea: The complete stop (> 90%) of the naso-oral airflow during a minimum of 10 seconds. The apnea was then classified as obstructive if it was accompanied by thoracic and abdominal effort, central if this effort was absent and mixed if both situations occurred in one single apnea.

- Hypopnea: A drop in the respiratory signal between 30% and 90%, accompanied by a drop in oxygen saturation ≥ 3% and/or arousal.

* Secondary outcomes:

Sleep characteristics: the total sleep time, the time of the partial phases of sleep (N1, N2 and N3) and the REM phase were calculated from the PSG study. The sleep fragmentation was measured by the arousal index.

Oxygen saturation: Cutaneous pulsioximetry with a finger probe (Palco laboratories P-340) was used to measure this variable during the performance of the PSG study. This variable was described by the mean oxygen saturation, minimum oxygen saturation and the percentage of time spent at SaO2 below 90% (CT90).

Snoring: On one hand both patient and bed mate/roommate were asked to evaluate the patient’s roncopathy by answering a questionnaire after finishing the treatment with each device. The question was How is your snoring? The possible answers were 1. Have increased a lot, 2. Have increased slightly, 3. Have not changed, 4. Have decreased slightly, and 5. Have decreased a lot. On the other hand, validated respiratory polygraphy system (Mesam IV) was used for the objective evaluation of the snoring habit (2). This system had the capacity to save information of 18 hours of recording. The snoring was measured by a protected microphone placed at the yugulum. The microphone filtered the sounds between 50 y 800 cycles/second as snoring sounds occurred in this range. If the volume of the sound is higher than 50% of the total volume, the sound was then identified as snore. If the sound exceeded a threshold of > 1.1 mV and 1000 cycles/second, it was then considered as strong snore. The data was processed by a personal computer and could be printed.

The system permitted an automatic or manual reading of the recordings in periods of 10 minutes. The study with Mesam IV was performed before treatment and after the 12 weeks of treatment with each device (MAD and PD). The snoring was described by measuring the intensity, number of snores per hour of recording, percentage of time of recording with evidence of snoring and its relationship with body postures during sleep.

Somnolence: the Epworth scale was used to measure the somnolence. The questionnaire also included other questions: Comparing to the situation before treatment how are you during the day? Comparing to the situation before treatment how is your humor? The roommate was specifically asked about his sleep in comparison to the he/she had before treating the patient. The answer for all these questions was selected from the following options: 1. Much worse; 2. Slightly worse; 3. No changes; 4. Slightly better; and 5. Much better.

Treatment compliance: it was evaluated by calculating the time of device use. This time was obtained from the patient´s declaration and in a consensus with the roommate. If there was no consensus, the patent’s declaration was considered as valid. Since the beginning of the study, patients had been phone called once per month to evaluate the treatment progress and patients adherence to the treatment. The use of the device was determined by the number of nights per week and the average number of hours of use per night since the last control. The average time of use of the device per hour was estimated by the multiplication of both variables and then the result was divided by 7. However, if the patient had used the device for different time between weeks, an average was calculated per each week and then the results were used to calculate the mean. It was considered a good adherence to the treatment if the mean time of use was ≥ 4 hours per night as described by the study of Ferguson *et al*. (20).

Complications: the nature, onset, duration, severity, and the outcomes of all adverse events, as well as any association of an adverse event related to the device (MAD and PD) were assessed and documented. In order to evaluate the safety profile of the treatments, all complications and/or adverse events were recorded with an accountability scale.

* Sample size calculation

The sample size was determined by taking in consideration the crossover design of the study. The objective was to be able to detect the minimum effect of the device on the variables of percentage of patients with AHI > 5, the distribution of AHI, the distribution of number of snores per hour of sleep and the percentage of patients with snoring. The calculation was performed considering α value of 0.05, statistical power (1-β) of 90% and bilateral hypothesis test.

A sample size of 40 subjects would result in a statistical power higher than 90%. In the case of AHI, a sample size of 40 patients permitted detecting a minimum difference of 9.5 in agreement with the data reported by Clark *et al*. (21) and Ferguson *et al*. (20) giving a standard deviation of 18 and the application of paired t-student test. In the case of number of snores per hour of sleep objectively measured in bed, a sample size of 40 patients permit detecting a minimum difference of 12.4 giving a standard deviation of 24.1.

* Statistical analysis 

The t-student test was used to compare the characteristics between groups. Paired t-student and analysis of variance were used for the comparison of continuous variables of paired samples. McNemar’s test was applied to see if the devise use had am effect over the AHI. Linear and multiple regression analysis were used for the crossover design and to examine the relationship between variables. The evaluation of the results was performed considering the potential effect of the period and the results were expressed for intention to treat and for every protocol.

## Results

Figure 1 describes the study flow chart. During a recruitment period of 6 months, 112 patients were evaluated. Sixty-three patients were excluded as they did not complete the inclusion criteria or had one or more of the exclusion criteria. Seven patients refused to participate in the study. Forty-two patients participated in the study and 38 patients (90.5%) completed all phases of the study. Four patients (9.5%) (2 men and 2 women) abandoned the study due to intolerance and/or secondary effects of the device.

 Table 1 shows the patients characteristics at the different phases of the study (adaptation, treatment with MAD and treatment with PD). Patients had a mean age of 46 ±9 years and 33 (79%) were males. There was no statistically significant difference in relation to the anthropometric characteristics and the clinical variables between the different phases of the study. However, a discrete increase in the mean body mass index was detected between the adaptation and placebo phases. This increase was from 27.7 ±3.2 to 29.3 ±9.2 (*p* < 0.05). The MAD achieved a mandibular protrusion of 8.6 ±2.8 mm. It was notorious the decrease in alcohol consumption between the adaptation and treatment phases for both study arms (*p* < 0.05).

**Table 1:**
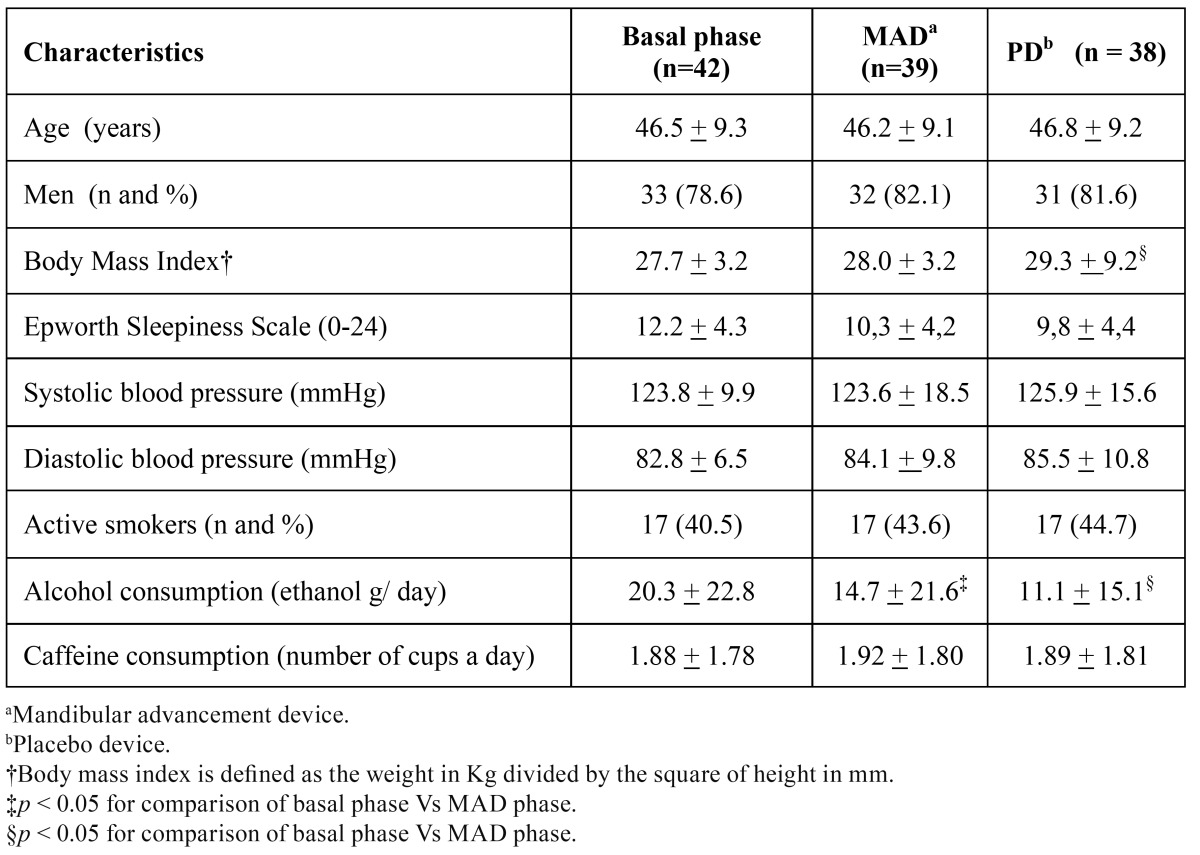
Characteristics of the study population.

 Table 2 shows the results of PSG at the different phases of the study. Neither MAD nor PD modified significantly the sleep duration, intensity or efficacy. Moreover, the comparison between the adaptation phase and the treatment phase in both study arms (MAD and PD) showed the absence of statistically significant differences in the total sleep time, the time of the partial phases of sleep (N1, N2 and N3) and the REM phase. The sleep fragmentation, measured by the arousal index, was not reduced by the treatment with MAD. However, it was significantly increased by the treatment with PD.

**Table 2:**
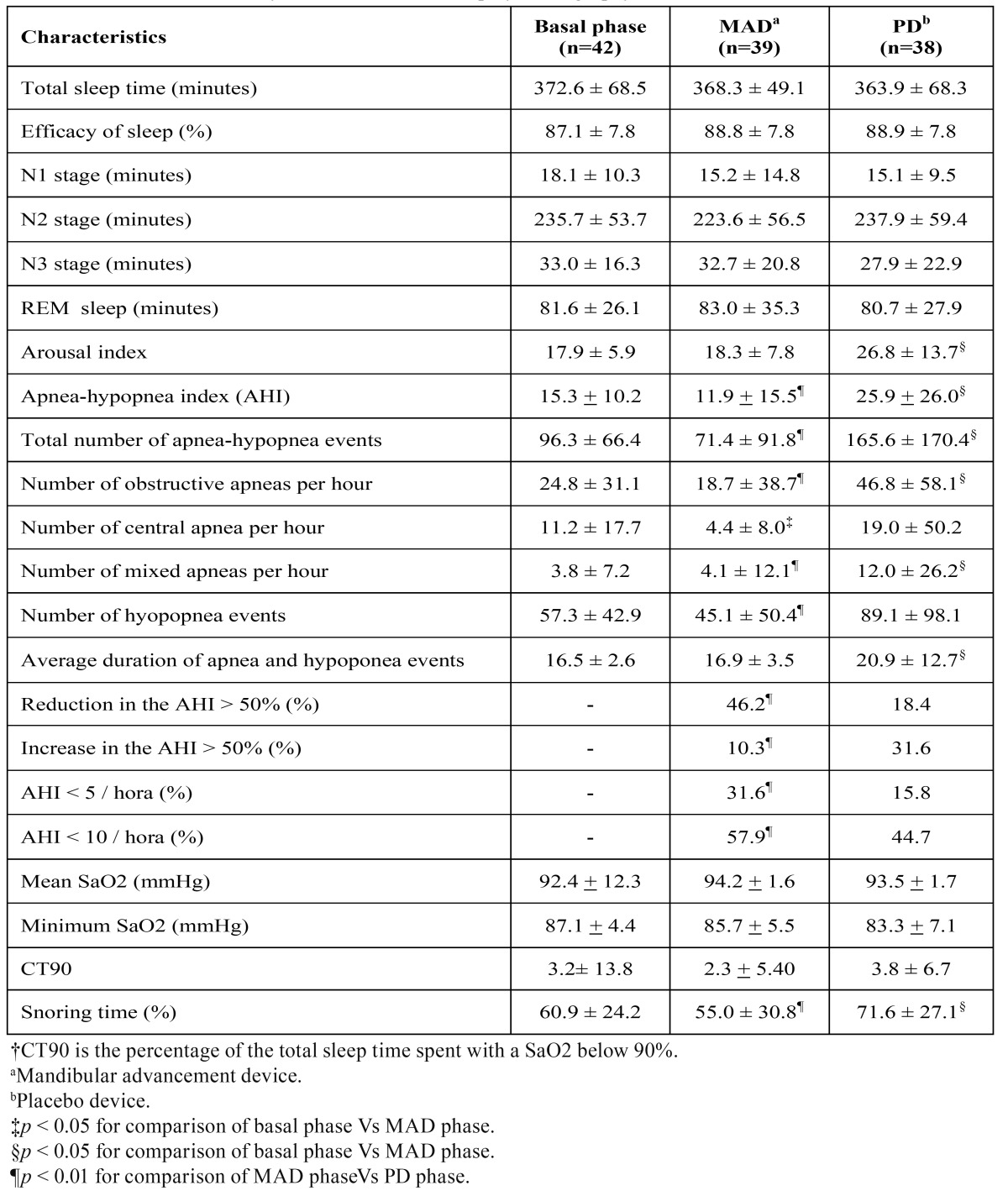
Outcomes of the analysis of the conventional polysomnography (PSG).*

The analysis of the respiratory events showed that MAD reduced the AHI from 15.3 +10.2 to 11.9 +15.5. This reduction was not statistically significant (*p* = 0.196). Whereas, the PD incremented the AHI to 25.6 + 26.0 and this increase was statistically significant (*p* = 0.016). Worth to mention, the use of MAD significantly reduced most of the respiratory events when compared to the PD as shown in  Table 2. For example, the 50% reduction in the basal AHI was achieved in 46.2% of the cases treated with MAD in comparison to the 18.4% for the PD. Values of AHI < 5 was achieved by MAD in the 31.6% of the cases in comparison to the 15.8% for the PD. There were no statistically significant differences when the variables of mean oxygen saturation, minimum oxygen saturation and CT90 were compared between the adaptation period and device-using phases.

The period effect was also evaluated by comparing the mean difference in the AHI of the patients initially treated with PD and the patients initially treated with MAD. The statistical analysis showed no significant differences. Additionally, the carryover effect and the interaction between the treatment and the period were also evaluated using the mean AHI after the use of MAD and PD. The results also showed the absence of a significant differences (data not shown).

 Table 3 describes the evolution of the roncopathy for the arms of the study. The evaluation was performed subjectively (questionnaire) and objectively (Mesam IV). The use of the MAD resulted in a significant reduction in the perception of snoring and increases the perceived quality of sleep when evaluated by both the patient and the bed mate/roommate. However, the objective evaluation showed no significant changes in the snoring characteristics.

**Table 3:**
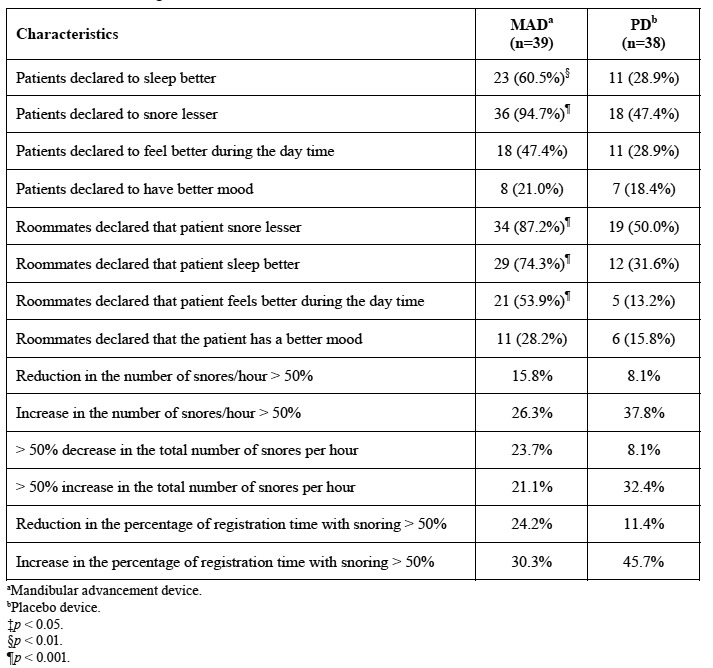
Results of snoring evaluation.

The compliance with the use of device was 6.4 + 2.4 hours for the MAD and 6.2 + 2.0 for the PD (*p* > 0.05). The 87.1% and the 76.3% of the patients used the MAD and PD respectively for more than 5 hours/night ( Table 3).

The secondary effects from the use of both splints are shown in table 4. Both splints induced the occurrence of relevant secondary effects (86.8% for the PD and 85.7% for the MAD). The 52.6% and 57.1% of these effects were mild for the PD and MAD, respectively. Severe secondary effects (irreversible alteration of the occlusion) were only observed in 5 patients due to the use of MAD.  Table 4 also showed that the secondary effects tended to be higher when the MAD was used.

**Table 4:**
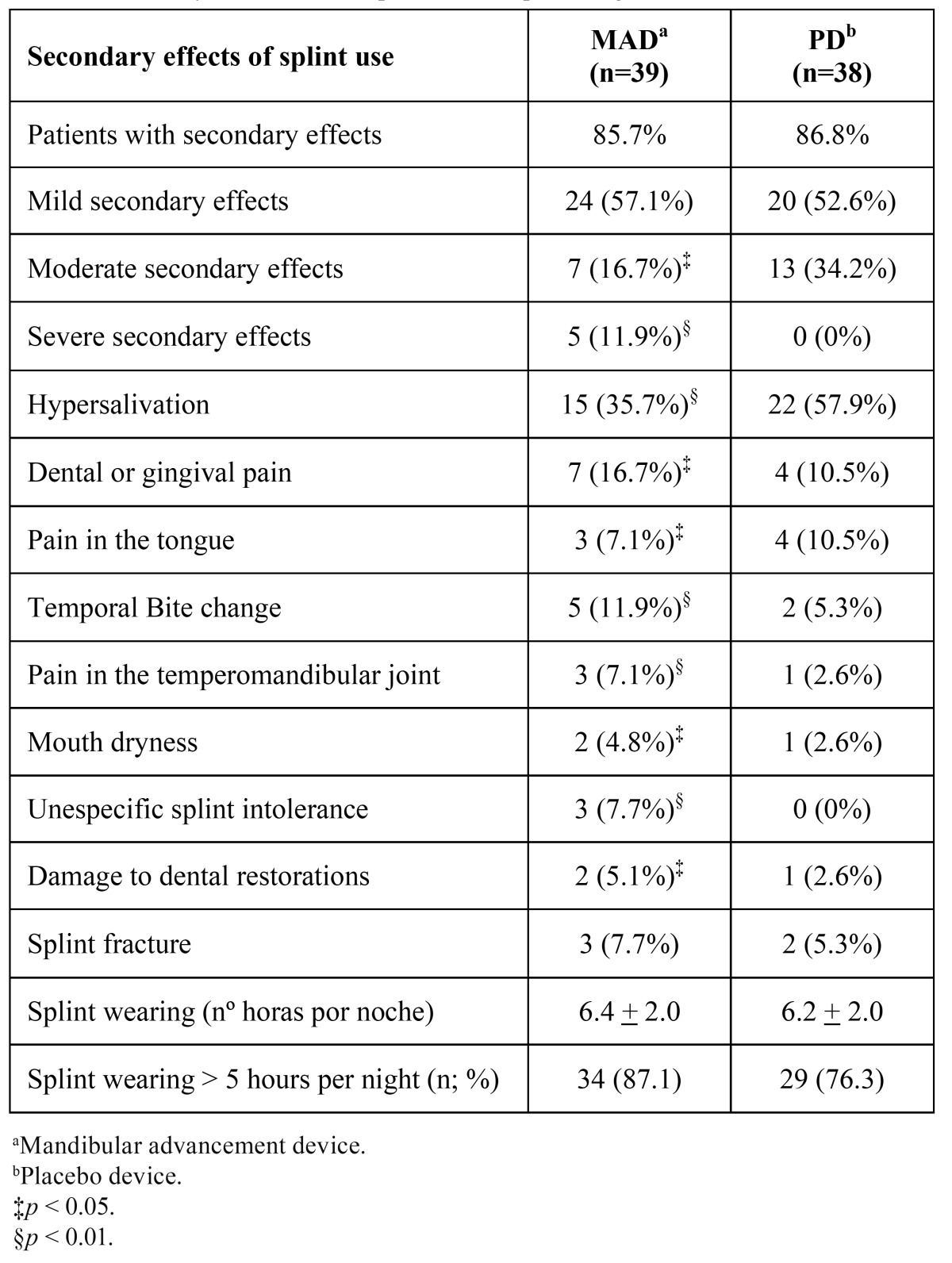
Secondary effects and compliance with splint usage.

**Figure 2:**
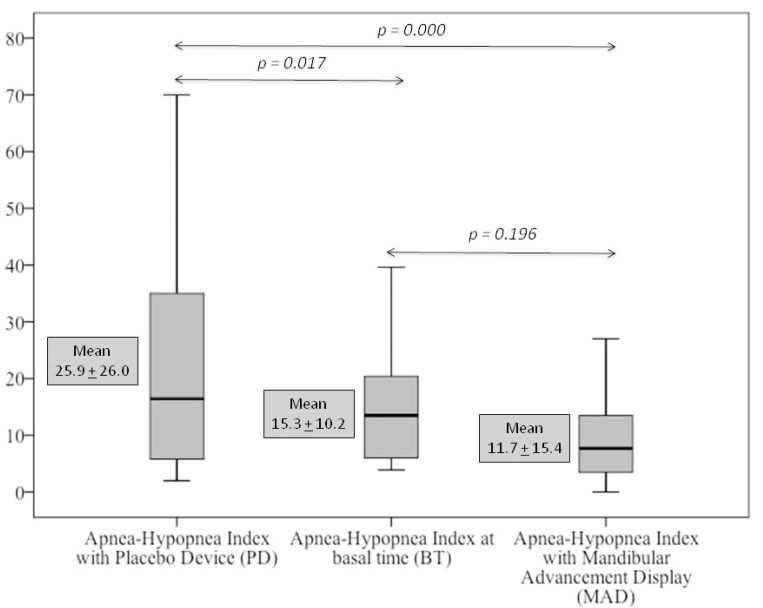
Effects of the device on the apnea-hypopnea index (AHI). Basal time (BT), placebo device (PD) and Mandibular Advancement device (MAD).

**Figure 3:**
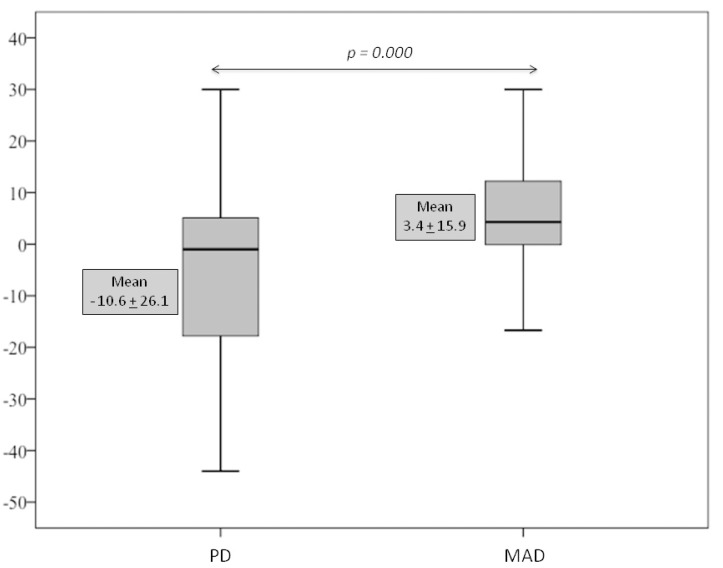
Effects of the device on the net change in the apnea-hypopnea index (AHI). The results were calculated as the difference between the AHI at basal time and the AHI after the use of the device. Placebo device (PD) and Mandibular Advancement device (MAD).

Figure 2 shows the comparison of the mean AHI between the basal phase with the PD and MAD phases. This comparison revealed a significant increase in the AHI by the PD (*p* = 0.017) and a reduction in the AHI, although not statistically significant, was observed in the MAD phase. When both splints were compared, the use of MAD reduced significantly the AHI (*p* = 0.000). Figure 3 shows the results of the mean change in the AHI. An increment in the AHI by 10.6 + 26.1 was caused by the PD while a reduction by 3.4 + 15.9 was caused by the MAD. The differences between the PD and the MAD were statistically significant ( *p* = 0.000).

## Discusion

The principal finding of this study is that mandibular advancement device, in comparison to a PD, has produced a significant enhancement in all the parameters that measure the respiratory events. As a secondary outcome, the MAD has reduced significantly the chronic roncopathy when evaluated by the bed mate/roommate. However, this reduction was not significant when the snoring was objectively measured.

In the last years several review studies have been published to evaluate the use of the MAD in the treatment of OSA (17-22). All these studies have concluded that this device has a positive effect in reducing the AHI but is inferior to the CPAP treatment (22). For that, there is no doubt that MAD is a reasonable alternative to the CPAP in patients with mild-to-moderate grade of OSA and in patients with severe OSA intolerant to the CPAP (22).

Although there are numerous clinical trials on the efficacy of MAD in patients with OSA, some issues remain unresolved. It is difficult to predict the type of patients who could maximally benefit from the use of MAD by having the highest tolerance to the splint and the least number of secondary effects (22).

Herein, when compared to PD, the MAD has significantly improved all respiratory parameters. The device reduced the absolute number of apnea and hypopnea as well as the AHI. The decrease in the AHI by more than 50% was achieved in almost half of the patients treated with MAD. AHI < 10 and AHI < 5 were achieved for the 57.9% and the 31.6% of patients treated with MAD and PD, respectively. Similar results have been reported by other controlled clinical trials (20,21) confirming the efficiency of MAD in the treatment of mild-to-severe OSA.

The selection of the type of the mandibular advancement device has been evaluated by several studies. Similar results have been reported by clinical trials that use two-piece design of the splint and trials that use mono-block designs (23). Recently, adjustable MAD (805 patients) and fixed (203 patients) have been compared in a retrospective study. The improvement in the OSA was better for the adjustable device than the fixed device (56.8% Vs 47.0%) (24).

Herein, an adjustable MAD was used to achieve the maximum and tolerable mandibular advancement (a minimum of the 65% of the maximum mandibular protrusion). This was adapted to reduce the number and/or intensity of the adverse events, minimize the injury to the temporomandibular joint and obtain better outcomes. A dose-response effect has been reported for the MAD, the more advancement of the mandible better is the response (25). In this study, the mean mandibular advancement was 8.8 + 2.8 mm.

Although it has been reported that when 50% of the maximum protrusion is achieved, there is a tendency for the secondary event to increase. In our study, 65% of the maximum protrusion has been achieved and the percentage of secondary effects has been similar to other studies (20,22).

One of the consequences of OSA is the loss of sleep architecture; lesser proportion of profound sleep (N3) and REM, and increase in the arousal index as expression of sleep fragmentation (3). The findings of this study indicated the absence of differences in the sleep characteristics, which could be related to the mild-to-moderate grade of OSA. Singh *et al*. (26) have found significant reduction in the arousals in OSA patients treated with MAD but the patients in that study had more severe sleep fragmentation (50.8 + 31.0) than our study. Regarding the oxygen saturation, the mild-to-moderate severity of OSA could also contribute to the absence of significant effect of the MAD (27).

Some studies have reported that the MAD produces a reduction in the values of arterial tension and improvements in the excessive daytime sleepiness (22). In our study, the MAD did not significantly alter these parameters nor the PD. Possibly, the severity of OSA (mild to moderate) could contribute to this finding.

With respect to the roncopathy, different studies have reported relevant improvements when MAD is used (28). The subjective evaluation of the snores has indicated a relevant improvement (about 90%). However, the objective evaluation has only shown mild and not significant improvements. This discrepancy could be attributed to the differences in the tolerance to the snores. During the objective evaluation, the system evaluates the presence/absence of snore but not its intensity. This means that a snore may persist but the reduction in its intensity would make it tolerable to the patient and the roommate/bedmate. However, Adriana *et al*. (27) have found improvements in the objective evaluation of the snores. This could be attributed to the fact that the basal situation of the patients in the study by Adriana *et al*. (27) was higher than the patients in our study and thus had more room for improvement. The snores/hour index was 40.4 and 24.5, respectively.

The secondary effects due to the use of the MAD were frequent. These effects were present in the 86% and 87% of the patients treated with MAD and PD, respectively. Although there were no significant differences between the groups, these secondary effects tend to be more severe in the MAD group.

Most of the secondary effects were mild to moderate. They were a subjective complaints of hyper salivation, mouth dryness, gingival pain, dental pain, lingual pain, or TMJ pain. This is in agreement of the findings of previous studies (22). However, severe secondary effects, referred to as an irreversible alteration of the occlusion, occurred in 5 patients due to the use of MAD. There was no association between the subjective complaints of the patients and the severe secondary effects. Data from the literature could suggest that irreversible occlusal alterations are related to the time of use of the MAD (21). This suggestion could not be evaluated in the study as the follow-up time was limited to 12 weeks in each arm of the trial.

One of the aspects related to the use of the MAD is the relative incapacity to predict which patients would benefit from the use of MAD and which not. It seems there is an individual variation in the response to a treatment with MAD (22). Some variables have been observed to be associated with the best response to a treatment with MAD. Of these variables are lower severity of the AHI, postural OSA, lower age, feminine gender, and lower obesity (22). Due to the relatively small sample size, inherent to a clinical trial, it was impossible to identify variables with a capacity to predict the response to the MAD. It is possible that the new techniques of image evaluation of the upper airway play a role in identifying patients that respond well to a treatment with MAD. This should be considered as priority in the clinical research in this field (29). Even is more intriguing the fact that in some patients if treated with MAD the AHI is worsened. The findings of this study indicated that 10.3% of the patients with MAD had their AHI increased by 50%, in comparison to the 31.6% in the case of the PD. This has been also observed in other studies (30). The etiology of such a response to the MAD is not totally understood and has been related to anatomical changes particularly in the position of the hyoid bone. This would make emphasis in the importance of performing a sleep study to monitor the outcomes of a treatment with MAD.

This randomized clinical trial has recruited 38 patients, a sample size that has been adequate to meet the study objectives. However, this sample size did not allow for patients sub grouping to identify factors that could be associated with a good response to the treatment with MAD. The criterion of maximum mandibular advancement, that was limited to a minimum of 65% of the maximum mandibular protrusion, was arbitrarily established. It is possible that lesser mandibular advancement could obtain similar results but with fewer secondary effects. Future research would test this hypothesis and establish a criteria to maximize the benefits and minimize the secondary effects. An important limitation to this study is that the used PD has not been a truly placebo (the results of the sleep study were affected by the PD). This limitation should be considered when analyzing the results of this clinical trial.

Within the limitation of this study, the use of MAD is efficient to reduce the AHI y improve subjectively the roncopathy. Thus, MAD could be considered in the treatment of mild-to-moderate OSA and chronic roncopathy. Further studies should evaluate the hypothesis of obtaining similar outcomes at minimal secondary effects when shorter mandibular advancement is achieved. Studies with larger sample size are still needed to establish the patient profile that could be associated with a good response to a treatment with MAD.
